# PATs and SNATs: Amino Acid Sensors in Disguise

**DOI:** 10.3389/fphar.2018.00640

**Published:** 2018-06-19

**Authors:** Shih-Jung Fan, Deborah C. I. Goberdhan

**Affiliations:** Department of Physiology, Anatomy and Genetics, University of Oxford, Oxford, United Kingdom

**Keywords:** SLC36A1, SLC36A4, SNAT2, SLC38A9, mechanistic target of rapamycin (mTORC1), transceptor, amino acid transporter

## Abstract

Solute Carriers (SLCs) are involved in the transport of substances across lipid bilayers, including nutrients like amino acids. Amino acids increase the activity of the microenvironmental sensor mechanistic Target of Rapamycin Complex 1 (mTORC1) to promote cellular growth and anabolic processes. They can be brought in to cells by a wide range of SLCs including the closely related Proton-assisted Amino acid Transporter (PAT or SLC36) and Sodium-coupled Neutral Amino acid Transporter (SNAT or SLC38) families. More than a decade ago, the first evidence emerged that members of the PAT family can act as amino acid-stimulated receptors, or so-called “transceptors,” connecting amino acids to mTORC1 activation. Since then, further studies in human cell models have suggested that other PAT and SNAT family members, which share significant homology within their transmembrane domains, can act as transceptors. A paradigm shift has also led to the PATs and SNATs at the surface of multiple intracellular compartments being linked to the recruitment and activation of different pools of mTORC1. Much focus has been on late endosomes and lysosomes as mTORC1 regulatory hubs, but more recently a Golgi-localized PAT was shown to be required for mTORC1 activation. PATs and SNATs can also traffic between the cell surface and intracellular compartments, with regulation of this movement providing a means of controlling their mTORC1 regulatory activity. These emerging features of PAT and SNAT amino acid sensors, including the transceptor mechanism, have implications for the pharmacological inhibition of mTORC1 and new therapeutic interventions.

## Transporters as Transceptors

**FIGURE 1:**
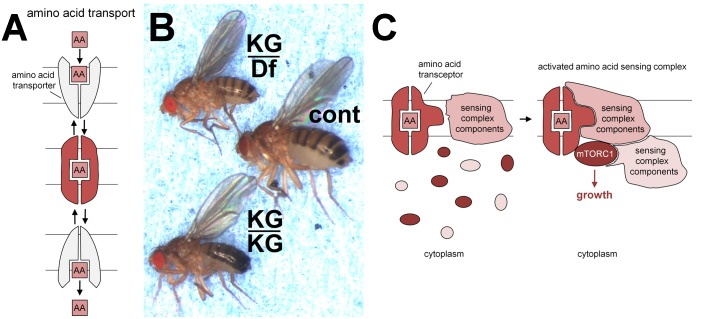
Transporters, pathetic flies and transceptors. **(A)** Schematic model of transporter switching between outward and inward facing conformations (upper and lower in gray), enabling molecules such as amino acids (AA) to be taken across lipid bilayers. The binding of such substrates is thought to stabilize an intermediate conformation (middle in red). Such transporters can function as symporters, co-transporting protons for some PATs and sodium ions for at least some SNATs. These ions determine the directionality of transport. **(B)** PAT amino acid transporter gene, *pathetic*, is required for normal growth *in vivo*. In comparison to normal controls (cont; middle fly) flies homozygous for the hypomorphic mutation, *pathetic^KG06640^* (KG/KG; lower fly) are small, or when heterozygous with a chromosomal deficiency (Df) removing the *pathetic* gene (KG/Df; upper fly) are even smaller. This small fly phenotype can be rescued by expression of a *pathetic* transgene. Mutations in other mTORC1 signaling pathway components also lead to small fly or small pupal phenotypes, for example, *S6 kinase* (Montagne et al., 1999) and *mTOR* (Zhang et al., 2000). This panel is reproduced with permission from the journal Development (Goberdhan et al., 2005). **(C)** Schematic model showing how PATs and SNATs may act as transceptors to activate mTORC1. Note that the molecular mechanism by which PATs and SNATs act as transceptors is currently unknown. The binding and/or translocation of amino acids or other substrates by PAT and SNAT transporters presumably induces specific conformational changes that generate a signal. Here, for illustration, we assume this signal is transmitted in the intermediate conformation (red; **A**), which may be formed during the transport cycle. The transceptor conformation facilitates the recruitment of membrane-bound sensing complex components (already assembled in a complex; Zoncu et al., 2011), cytoplasmic sensing complex components and mTORC1 constituents. This leads to the formation of a functional sensing complex that can respond to amino acid signals from the lumen of the intracellular compartment (where extracellular amino acids can rapidly accumulate; Zoncu et al., 2011) to activate mTORC1 signaling and drive cell growth.

Many nutrients are shuttled across the plasma membrane into the cytosol by transporters. The presence of nutrients can, however, also often be detected on the outside of the cell by classical signal-transducing receptors. The concept of overlap between these groups was highlighted by the discovery in yeast of transporter-related receptors, now known as transceptors, which have now been identified in a range of higher organisms (reviewed in Thevelein and Voordeckers, 2009). In some cases, transceptor function appears to be linked to transport ability, but in others, only binding, and not transport, may be necessary (see also **Figure 1C**). Below we review the evidence that members of the PAT (SLC36) and SNAT (SLC38) amino acid transporter families act as transceptors to control mTORC1 signaling and that this takes place on intracellular membranes.

## Specific Solute Carriers Transport Amino Acids and Control mTorc1-Mediated Homeostasis

Solute Carriers (SLCs) are transmembrane transporters required to maintain intracellular homeostasis through their ability to translocate small soluble molecules such as nutrients, drugs and waste products across lipid bilayers. SLCs are secondary active or facilitative transporters, employing a second substrate or an electrochemical gradient, respectively, to drive transport. They consist of a central pore and gating system that allows the passage of substrates in a regulated manner via conformational changes rather than the opening of a channel (**Figure 1A**).

The SLC superfamily has been divided into over fifty SLC families (Perland and Fredriksson, 2017), based on sequence homology. Members of individual families often have properties in common. One of these is their mechanism of transport, for example, whether they transport their substrate in symport with specific ions. Another is that their substrates, for example, nutrients, metabolites or drugs, have similar chemical features. It has been estimated that over 25% of SLCs transport amino acids as their primary substrates (Fredriksson et al., 2008). Some have broad amino acid specificity (see Devés and Boyd, 1998), including CD98 heterodimeric transporters and SLC6A14 (Sikder et al., 2017). Others are highly selective, for example, the Proton-assisted Amino acid Transporter family (PAT or SLC36), with prototypical substrates, alanine, glycine and proline, and the Sodium-coupled Neutral Amino acid Transporter (SNAT or SLC38) family, which co-transports small neutral amino acids, such as alanine, glutamine, serine, glycine, methionine, and threonine together with sodium ions. In addition to acting as building blocks for protein synthesis, intracellular amino acids are potent activators of a major signaling cascade, which controls this very same process, involving mechanistic Target of Rapamycin Complex 1 (mTORC1). mTORC1 is also activated by growth factor signaling and promotes mRNA translation by increasing the number and activity of ribosomes to stimulate cell growth and proliferation (Goberdhan et al., 2016). While early studies implicated leucine as a major mTORC1 activator (Hara et al., 1998; Christie et al., 2002; Beugnet et al., 2003), other amino acids are now known to be involved, including arginine (Wang et al., 2015; Carroll et al., 2016), glutamine and serine (Jewell et al., 2015; Rebsamen et al., 2015; Fan et al., 2016).

## Pat Amino Acid Transporters Control Growth and mTorc1 Signaling

In 2005, an *in vivo* genetic screen was undertaken in the fruit fly, *Drosophila melanogaster*, by overexpressing amino acid transporters from a broad range of SLC families, to identify positive regulators of mTORC1 activity and growth (Goberdhan et al., 2005). Members of the PAT (SLC36) family were highlighted as particularly potent growth-promoting transporters.

This was curious, because the rationale of the screen had been to identify cell surface transporters that deliver amino acids to intracellular mTORC1, but the known properties of the two characterized mammalian PATs, PAT1 (SLC36A1), and PAT2 (SLC36A2) were at odds with this idea. On account of its subcellular localisation in rat brain tissue, the first mammalian PAT identified was named lysosomal amino acid transporter 1 (Lyaat-1; Sagné et al., 2001), now renamed PAT1. Furthermore, PAT1 and PAT2 transport amino acids in symport with protons, a mechanism that favors transport from the acidic lumens of late endosomes and lysosomes (LELs) into the cytosol. In fast-growing cultured human cells, PAT1 co-localizes with LAMP1, a marker of LELs (Ögmundsdóttir et al., 2012). GFP-tagged fly PATs are also predominantly located intracellularly. These characteristics raised the question of whether PATs might act to promote growth from inside cells (reviewed in Goberdhan et al., 2009).

Two fly PATs, CG1139 and CG3424, were highlighted in the genetic screen; they share sequence similarity with all members of the human PAT family (Goberdhan et al., 2005). Adult flies carrying a mutation in the ubiquitously expressed *CG3424* were growth-retarded (**Figure 1B**). As is the convention in the *Drosophila* field, *CG3424* was renamed *pathetic* (*path*) to reflect this mutant phenotype; this also reflected the transporter’s properties (PAT with high affinity; see below). Amino acid levels in fly food are likely to modulate the effects of sensors on mTORC1-associated growth. Indeed, the Parrish group has reported that flies homozygous for the same *path* mutant grow to normal size under their culture conditions. They did, however, show that *path* is required for the growth of large, but not small, dendrites in flies, highlighting a selective requirement for Pathetic under these conditions, when exceptional levels of growth are needed (Lin et al., 2015).

Genetic experiments in which both *path* and *CG1139* overexpression are combined with changes in mTORC1 signaling components suggest these transporters activate mTORC1 (Goberdhan et al., 2005). Human PAT1 and PAT4 (SLC36A4), which are both ubiquitously expressed, also promote cell proliferation and mTORC1 signaling, based on genetic knock down and overexpression in cultured human cells (Heublein et al., 2010; Fan et al., 2016). Both can also induce growth and modulate mTORC1-dependent functions when overexpressed in flies (Heublein et al., 2010).

It is worth noting that in contrast to these findings, overexpression of PAT1 has been shown by the Sabatini group to reduce mTORC1 signaling (Zoncu et al., 2011). Although they suggested that amino acid depletion from the lysosome might be responsible, the restricted substrate specificity of PAT1 for alanine, glycine and proline transport does not support this view. An alternative explanation is that high levels of PAT1 produce a type of ‘dominant negative effect’, by interfering with the assembly of complete PAT-mTORC1 growth-promoting complexes. Such a phenomenon was reported by Goberdhan et al., 2005, where overexpressing one copy of a PAT causes increased growth in the wing *in vivo*, while two copies reduce growth (see Supplementary Figure 1 and further discussion in Goberdhan et al., 2016). Overexpression of abnormally high levels of the *Drosophila* homolog of mTOR has also been shown to inhibit growth. This was proposed to be due to the high levels of mTOR binding and titrating away factors essential for normal mTOR function, so that assembly of complete functional complexes was reduced (Hennig and Neufeld, 2002). These experiments emphasize the importance of controlling PAT transporter levels when assessing effects on mTORC1 and growth, suggesting that simple transport may not underpin the growth regulatory properties of these molecules.

## Pat and Snat Transceptors Can Regulate mTorc1 Signaling

The transport characteristics of fly PAT CG1139 are very similar to those of characterized mammalian PATs when expressed in the heterologous *Xenopus* oocyte system (Goberdhan et al., 2005). PATH, however, behaves very differently. It has a much higher affinity for alanine than CG1139, coupled with much lower transport capacity. Indeed PATH was predicted to increase bulk intracellular amino acid concentration by less than 0.2% in oocyte experiments. Despite this, PATH activated S6-kinase, an established mTORC1 target, in this system. These findings suggested that PATH might be acting as a transport-independent transceptor (**Figure 1C**; Goberdhan et al., 2005).

PATH’s poor ability to transport amino acids raised the possibility that additional amino acid transporters might control mTORC1 via a transceptor mechanism. Attention focused on human PAT4, which at the time had no known transport substrates. PAT4 was found to exhibit very high substrate affinity and low capacity for proline and the atypical PAT substrate tryptophan, when expressed in *Xenopus* oocytes (Pillai and Meredith, 2011). PAT4 was also found to transport in an electroneutral manner. These properties were in keeping with the transport characteristics of PATH and led to the suggestion that PAT4, which activates mTORC1 in human cells (Heublein et al., 2010), may be a functional equivalent of PATH (Pillai and Meredith, 2011). Alternatively transceptor function may be shared by all members of the PAT family, irrespective of their transport capacity.

Other researchers interested in uncovering the link between amino acid transporters and mTORC1 focused on a member of the SNAT family of transporters called SNAT2 (SLC38A2), and approached the identification of transceptor function from a different angle. There are eleven members of the human SNAT family, although the transport properties of only some of them have been studied in detail (Schiöth et al., 2013). Elegant experiments involving the transport of the non-metabolisable SNAT2 substrate MeAIB (α-methylaminoisobutyrate) demonstrated that this compound could activate mTORC1 signaling via SNAT2 during amino acid starvation. Under these specific conditions, MeAIB increased cell proliferation in MCF7 breast cancer and L6 myotubes (Pinilla et al., 2011). Substrate binding and transport alone, therefore, is sufficient for SNAT2 to activate mTORC1, even if the substrate cannot subsequently be utilized in the downstream metabolic pathways that normally act on transported amino acids.

PAT and SNAT families are closely related in their eleven putative transmembrane domains (Schiöth et al., 2013). This may explain why members of both families have transceptor function. Currently the mechanism by which amino acid interaction with PATs and SNATs leads to transceptor activation remains unclear. Presumably, amino acid binding and/or translocation could lead to conformational changes in the transporter and/or altered interaction with an associated protein complex, which then increases mTORC1 signaling (**Figure 1C**).

## An Amino Acid Sensing Complex on Late Endosomes and Lysosomes Controls mTorc1 Activity

Reports identifying the human PATs as mTORC1 regulators emerged shortly after their established sites of action, the LELs, were shown to be key places where mTORC1 signaling is controlled by amino acids via the Rag GTPases, central components of an amino acid sensing complex required for mTORC1 recruitment and activation on these compartments (Sancak et al., 2008). Over the last 10 years the number of components identified in this sensing complex has increased, including membrane-associated components, such as the v-ATPase proton pump. In addition, a range of cytosolic amino acid and glucose sensors has been proposed, including Leucyl-tRNA synthetase (Han et al., 2012), Castors, Sestrins, TSC/Rheb (reviewed in Wolfson and Sabatini, 2017) and aldolase (Lin and Hardie, 2018).

The presence of an amino acid sensing complex spanning the surface of intracellular compartments raised the possibility that it also sensed nutrients from the luminal face. The LEL lumen is topologically equivalent to the extracellular milieu and so could provide a read-out of the extracellular environment. Indeed, Zoncu et al. (2011) have shown that labeled extracellular amino acids enter the LEL lumen within 10 min. Amino acid transporters that span the lipid bilayer and can bind amino acids seemed ideal candidate sensors.

## Late Endosomal and Lysosomal Pats and Snats Regulate mTorc1 Signaling

**FIGURE 2:**
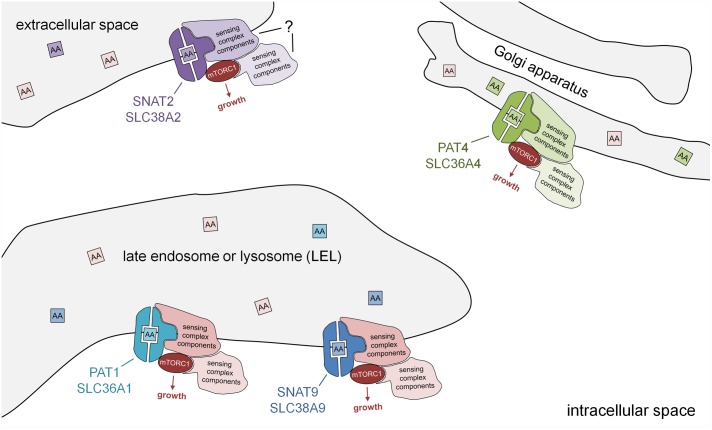
Subcellular localisation of PAT and SNAT transporters: amino acid sensors in disguise. Schematic model showing PAT and SNAT amino acid transceptors, which can respond to specific amino acids in the lumen of intracellular compartments by activating mTORC1 and growth via a transport-independent mechanism. Note that the molecular mechanism by which PATs and SNATs act as transceptors is currently unknown. PAT and SNAT transporters have been identified in several different locations in cells, namely the plasma membrane, acidic late endosomes and lysosomes (LELs), and also the Golgi apparatus. In the transceptor model, the binding and/or translocation of specific amino acids or other substrates induces a conformational change that alters interactions with associated proteins and increases mTORC1 signaling. The amino acid sensing complexes associated with SNAT2 have not yet been characterized. Different transporters have been shown to shuttle between compartments; for example, in human cells, PAT1 (plasma membrane and LELs) and SNAT2 (Golgi and plasma membrane), thus modulating their mTORC1-regulatory activity.

Human PAT1 was the first amino acid transporter located on LELs, which was shown to interact physically with the Rag GTPases and to be required for amino acid-dependent mTORC1 recruitment to these compartments. It was proposed that it was part of a sensing assembly that detects amino acids in the luminal space of these compartments to activate mTORC1 (Heublein et al., 2010; Ögmundsdóttir et al., 2012; **Figure 2**).

More recently, a SNAT, SLC38A9, has also been shown to localize to LELs and to strongly interact with the Rag GTPases and other components of the amino acid-sensing mTORC1 super-complex via its cytosolic N-terminal region. This N-terminal interaction with the Ragulator and Rags is, however, insensitive to amino acids (Jung et al., 2015; Rebsamen et al., 2015; Wang et al., 2015). Full-length SLC38A9 transports arginine with low affinity and also glutamine, but its arginine-dependent interactions with the Rags require much lower, more physiological levels of arginine (Wyant et al., 2017), supporting a transceptor function. A form of SLC38A9 that lacks the N-terminal region (SLC38A9.2) can still physically interact with the v-ATPase and is sensitive to amino acids, suggesting that the transmembrane regions in this molecule, which are the domains conserved between PATs and SNATs, are likely to be central to its amino acid sensing ability (Wang et al., 2015). Given the transceptor function attributed to other SNATs and some PATs, it seems likely that SLC38A9 shares a related mTORC1-regulatory transceptor mechanism.

Although the weight of evidence from different systems strongly supports the role of amino acid transporters in transceptor-mediated mTORC1 activation, the limitations of each experimental approach employed also needs to be considered. Transport assays are performed in a heterologous system, such as *Xenopus* oocytes or proteoliposomes, not *in situ*. Experiments in human cell culture are typically performed following complete amino acid starvation and may involve the addition of single amino acids; how this relates to physiological conditions remains unclear. This will be particularly relevant when substrates that bind at different affinities are mixed. Indeed, recent transport studies with SLC38A9 suggest a complex interplay between arginine and other amino acids, such as leucine, which it has now been shown to transport at high affinity (Wyant et al., 2017). *In vivo* approaches effectively circumvent this problem, but specific mutant transporters or substrates that could distinguish transceptor- and transporter-dependent mechanisms have yet to be developed.

## Golgi-Localized Pat4 Can Regulate mTorc1 Signaling

The Golgi apparatus provides a second site from which mTORC1 can be activated (Thomas et al., 2014; Jewell et al., 2015). Indeed, an analysis of PAT4 revealed that it is strongly localized to the *trans*-Golgi in cancer cell lines (Fan et al., 2016). Proximity ligation assays (Weibrecht et al., 2010) suggested that PAT4 interacts with Rab1A and mTOR at this location. PAT4 has two properties that distinguish it from the LEL-localized, mTORC1-regulating transporters. First, it has a transport preference for serine and glutamine, the latter being another key amino acid required for metabolism in many cancers (Zhang et al., 2017). Second, it seems to regulate a form of mTORC1 that has a stronger effect on one of its targets, the translation initiation inhibitor 4E-binding protein 1 (4E-BP1), than on another, the S6-kinase/ribosomal S6 pathway, unlike LEL-localized mTORC1. Given the observation that PAT4 is a high affinity, low capacity transporter, these findings suggest that it represents an alternative mTORC1-regulating transceptor on the Golgi (**Figure 2**).

It should also be remembered that other transceptors in the SNAT family that have been assumed to function at the cell surface, have the potential to act as intracellular transceptors. For example, SNAT2 is thought to act at the plasma membrane and to be stored in the Golgi (Hatanaka et al., 2006), when not required. It will be important to check that this Golgi-located SNAT2 is not involved in mTORC1 regulation in future studies.

## Regulation of Pat and Snat Activity Through Intracellular Shuttling

Another implication of the compartment-specific transceptor functions of PATs and SNATs is that their trafficking within the cell will significantly affect mTORC1-regulatory activity. Although the subcellular localisation of mammalian PATs is reported to vary between cell types (Sagné et al., 2001; Chen et al., 2003; Rubio-Aliaga et al., 2004), studies investigating the control of that localisation have been limited to overexpression experiments with GFP-tagged PATs in *Drosophila*. Oncogenic signaling through loss of the major tumor suppressor, PTEN, which blocks PI3-kinase signaling, leads to a striking shift in PAT localisation into intracellular compartments. This is accompanied by a synergistic enhancement in growth (Ögmundsdóttir et al., 2012; reviewed in Goberdhan et al., 2016).

It is tempting to speculate that several transporters in the PAT and SNAT families play a dual role: as transporters at the plasma membrane and transceptors inside the cell, with extracellular signals, such as nutrients or growth factors, determining the balance between these two functions. This could be particularly significant in cancer where growing cells must adapt to changes in their microenvironment that are typically not experienced in normal physiological conditions.

## Pats and Snats as Therapeutic Targets to Block Cancer Progression

The expression of a growing number of SLC transporters has been implicated in cancer progression, and there has been interest in targeting them to block cancer progression, for example, through effects on ‘glutamine addiction’ (reviewed in Bhutia et al., 2015) and in metastatic prostate cancer (Wang et al., 2013). PAT and SNAT family members are implicated in cancer progression. For example, SNAT1 and SNAT2 are required to support glutaminolysis in cancer cells (Bröer et al., 2016). In addition, high PAT4 expression is associated with reduced relapse-free survival after colorectal cancer surgery. A detailed analysis of PAT4 function in the colorectal cancer cell line HCT116 suggested that high expression of PAT4 leads to resistance to depletion of glutamine (Fan et al., 2016). Cells become more addicted to glutamine as their metabolism switches toward increased glycolysis, the so-called Warburg effect (Smith et al., 2016). Therefore, PAT4 may take on a more prominent function in some cancers as they come under nutrient stress, caused for example by reduced blood supply. The role of SLC38A9 in cancer is complex. It appears to be downregulated in colorectal cancer (Abdul Aziz et al., 2016). In pancreatic cancer, however, it has been reported to become essential when macropinocytosis, stimulated by the oncogenic form of K-Ras, becomes the primary mechanism by which tumor cells acquire nutrients, which are released by protein degradation in the lysosome (Wyant et al., 2017).

Overall, a picture is emerging in which multiple transceptors, which have enhanced function under certain microenvironmental conditions, are likely to be involved in cancer growth. This coupled with their potential synergistic interactions with oncogenic signaling, may provide new therapeutic opportunities to selectively target cancer cells, particularly in combinatorial treatments. Such developments will require a more detailed analysis of the conditions under which their function becomes essential, as well as a better understanding of the transceptor signaling mechanism.

## Uncovering the Pat and Snat Transceptor Mechanism

A number of research groups around the world have now highlighted different members of the PAT and SNAT families as amino acid-sensing transceptors that can activate mTORC1. The range of evidence and experimental models used strengthens the idea that PATs and SNATs share a common transceptor function. Several technical challenges, however, remain and mean we are some distance from understanding the mechanistic details.

A comprehensive analysis of the structural features in PATs and SNATs mediating transceptor function is needed. Of note is a threonine residue in the first transmembrane domain of SLC38A9, conserved in human and fly PATs as well as other SNATs. As this is essential for amino acid binding, it would also be required for transceptor function (Wyant et al., 2017). Mutations in residues such as this will be useful in blocking signaling or possibly altering amino acid specificity; dissecting the transceptor mechanism will, however, require an understanding of the conformational changes that amino acids induce.

A second priority is to screen for small molecules that can block transceptor function, with success dependent on the transceptor mechanism. This is illustrated by MeAIB, which competes with SNAT2′s amino acid substrates, but under amino acid starvation can activate its mTORC1-regulatory transceptor function (Pinilla et al., 2011). Finding an inhibitor of transceptor function will require the identification of a molecule that binds to the transporter, but prevents it from adopting the ‘transceptor conformation’ (**Figure 1C**).

Over more than a decade the study of PATs and SNATs has highlighted a new paradigm for transporter function, where these molecules not only function via the assembly of a signaling complex, but also can do this in specific intracellular compartments. Moving forward, we will see whether other nutrient transporters in humans have taken on similar roles during evolution, and whether they also have important roles in disease.

## Author Contributions

S-JF and DG have both made contributed intellectually to this work.

## Conflict of Interest Statement

The authors declare that the research was conducted in the absence of any commercial or financial relationships that could be construed as a potential conflict of interest.
