# Supplementary material for: PATs and SNATs: Amino Acid Sensors in Disguise
Source: Front Pharmacol. 2018 Jun 19;9:640. doi: 10.3389/fphar.2018.00640 (PMC6018406; doi:10.3389/fphar.2018.00640)
Supplement: Supplementary file 1 [file Image_1.pdf]

Supplementary Material

PATs and SNATs: Amino Acid Sensors in Disguise

Shih-Jung Fan and Deborah C. I. Goberdhan*

*** Correspondence:** Corresponding Author: deborah.goberdhan@dpag.ox.ac.uk

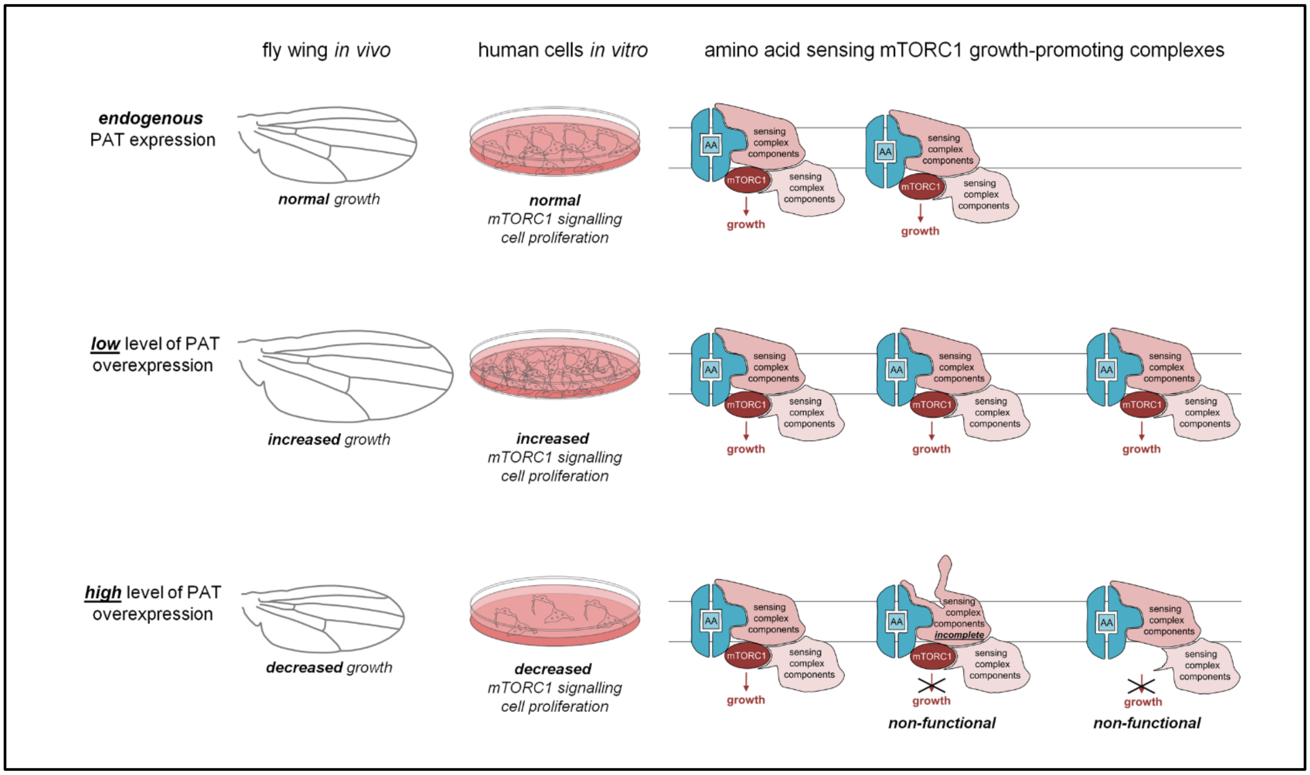

Supplementary Figure 1. Impact of PAT overexpression on functional PAT-growth-promoting complexes. Schematic model showing the effect of overexpressing a PAT amino acid transceptor, depicted in blue (bound to an amino acid substrate; AA), on the generation of PAT growth-promoting complexes. The molecular mechanism by which PATs may act as transceptors is currently unknown. *Endogenous* PAT expression promotes growth through amino acid sensing mTORC1 growth-promoting complexes in the fly wing *in vivo* and in human cell lines *in vitro*. *Low* levels of PAT overexpression leads to *increased* growth both in the fly wing (Goberdhan *et al*., 2005) and in human cells grown in culture (Heublein et al., 2010), presumably because the number of 'functional' growth-promoting complexes is increased. Abnormally *high* levels of PAT overexpression may, however, result in *decreased* growth (bottom row; Goberdhan *et al*., 2005; Zoncu et al., 2011), because proteins which bind to the PAT and are required to make a functional PAT growth-promoting complex, eg sensing complex components or mTORC1 are recruited to incomplete complexes, resulting in the formation of *non-functional* signaling units.
